# Clinical analysis on diagnostic accuracy of Bosch Vivalytic SARS-CoV-2 point-of-care test and evaluation of cycle threshold at admission for COVID-19 risk assessment

**DOI:** 10.1186/s12879-022-07447-7

**Published:** 2022-05-23

**Authors:** Lukas Andreas Heger, Nils Elsen, Marina Rieder, Nadine Gauchel, Urte Sommerwerck, Christoph Bode, Daniel Duerschmied, Mark Oette, Ingo Ahrens

**Affiliations:** 1 Department of Cardiology and Angiology I, Heart Center Freiburg University, Faculty of Medicine, University of Freiburg, Hugstetter Strasse 55, 79106 Freiburg, Germany; 2 Department of Cardiology and Medical Intensive Care, Augustinerinnen Hospital, Academic Teaching Hospital University of Cologne, Cologne, Germany; 3 Department of Pneumology, Augustinerinnen Hospital, Academic Teaching Hospital University of Cologne, Cologne, Germany; 4 Department of General Medicine, Gastroenterology and Infectious Diseases, Augustinerinnen Hospital, Academic Teaching Hospital University of Cologne, Cologne, Germany

**Keywords:** COVID-19, Point of care, PCR test, Length of stay, Patient flow

## Abstract

**Background:**

Point-of-care (POC) polymerase chain reaction (PCR) tests have the ability to improve testing efficiency in the Coronavirus disease 2019 (COVID-19) pandemic. However, real-world data on POC tests is scarce.

**Objective:**

To evaluate the efficiency of a novel severe acute respiratory syndrome coronavirus 2 (SARS-CoV-2) POC test in a clinical setting and examine the prognostic value of cycle threshold (CT) on admission on the length of hospital stay (LOS) in COVID-19 patients.

**Methods:**

Patients hospitalised between January and May 2021 were included in this prospective cohort study. Patients’ nasopharyngeal swabs were tested for SARS-CoV-2 with Allplex™2019-nCoV (Seegene Inc.) real-time (RT) PCR assay as gold standard as well as a novel POC test (Bosch Vivalytic SARS-CoV-2 [Bosch]) and the SARS-CoV-2 Rapid Antigen Test (Roche) accordingly. Clinical sensitivity and specificity as well as inter- and intra-assay variability were analyzed.

**Results:**

120 patients met the inclusion criteria with 46 (38%) having a definite COVID-19 diagnosis by RT-PCR. Bosch Vivalytic SARS-CoV-2 POC had a sensitivity of 88% and specificity of 96%. The inter- and intra- assay variability was below 15%. The CT value at baseline was lower in patients with LOS ≥ 10 days when compared to patients with LOS < 10 days (27.82 (± 4.648) vs. 36.2 (25.9–39.18); p = 0.0191). There was a negative correlation of CT at admission and LOS (r[44]_s_ = − 0.31; p = 0.038) but only age was associated with the probability of an increased LOS in a multiple logistic regression analysis (OR 1.105 [95% CI, 1.03–1.19]; p = 0.006).

**Conclusion:**

Our data indicate that POC testing with Bosch Vivalytic SARS-CoV-2 is a valid strategy to identify COVID-19 patients and decrease turnaround time to definite COVID-19 diagnosis. Also, our data suggest that age at admission possibly with CT value as a combined parameter could be a promising tool for risk assessment of increased length of hospital stay and severity of disease in COVID-19 patients.

## Contribution to the field

Coronavirus disease 2019 (COVID-19) is a rapidly spreading global pandemic with increased burden on healthcare systems. Often, centralised laboratory polymerase chain reaction (PCR) testing leads to long severe acute respiratory syndrome coronavirus 2 (SARS-CoV-2) test turnaround times, which in return result in poor patient flow and nosocomial transmission. Point-of-care (POC) PCR tests have the ability to improve testing efficiency by reducing steps between sample collection and results, ultimately contributing to better COVID-19 containment. Numerous assays are suggested for use in clinical practice. However, with some of them just recently released, data on their clinical performance is limited. We provide prospective real world data supporting the accuracy and reliability of a new rapid PCR point of care test (Bosch Vivalytic SARS-CoV-2) in a comparative analysis with an external laboratory performing RT-PCR as gold standard. Also our data suggests that at admission POC Cycle threshold (CT) might be a promising marker for length of hospital stay and possibly severity of disease in COVID-19 patients. Furthermore our data emphasise the increased risk for long-term health effects in COVID-19 patients and the need for individual follow-up.

## Introduction

Severe acute respiratory syndrome coronavirus 2 (SARS-CoV-2) with the resulting Coronavirus disease 2019 (COVID-19) pandemic has presented hospitals with considerable challenges [1]. These are partly aggravated by long COVID-19 test turnaround times, associated with centralised laboratory polymerase chain reaction (PCR) testing, resulting in poor patient flow and nosocomial transmission [2, 3]. Standard real-time (RT)—PCR for COVID-19 is the gold standard but requires specialized materials, equipment, personnel and transportation to a centralized laboratory [4]. Point-of-care (POC) PCR tests have the ability to improve testing efficiency by reducing steps between sample collection and results, ultimately contributing to better COVID-19 containment [3]. Numerous assays are suggested for use in clinical practice. However, with some of them just recently released, data on their clinical performance is limited [5]. We evaluate in a prospective clinical setting the Bosch Vivalytic (Bosch Healthcare Solutions GmbH; Stuttgarter, Germany) SARS-CoV-2 test for Bosch Vivalytic One and the COVID-19 ELISA Elecsys Anti-SARS-CoV-2 assay (Roche Holding AG; Basel, Swiss) with a centralised laboratory PCR test (Seegene [Seegene, Inc; Seoul, South Korea] AllplexTM2019-nCoV) carried out by an extern laboratory (Laboratory Dr. Wisplinghoff, Horbeller Str. 18-20 Koeln, Germany) as gold standard.

The Vivalytic analyser developed by Bosch is a portable device for molecular diagnostics, able to perform PCR procedures fully automatically. The increment has to be collected in a guanidine thiocyanate-based medium (eNAT^®^ [COPAN Diagnostics Inc.; Murrieta, USA)]), which stabilizes the viral RNA and completely inactivates the microbial viability. After that the composite is loaded into a specific Vivalytic SARS-CoV-2 cartridge. The Bosch Vivalytic SARS-CoV-2 is singleplex test, which targets the E gene sequence for COVID-19 detection. It takes 39 min to be performed on a specimen volume of 300 µl.

Current data point towards a plausible positive correlation between the amount of detected virus measured by the proxy PCR cycle threshold (CT) value and the severity of disease [6]. Previous evidence from influenza suggests that the higher the initial viral load, the worse the clinical development [7]. However, firm conclusion on the relationship between initial CT value and individual prognosis is still missing and data for the most part limited to nasopharyngeal viral load samples processed through external laboratories [8]. Whether CT values derived from POC testing could yield similar results is yet to be established.

Different scoring systems have been suggested for COVID-19 risk-assessment, with several of them being derived from established assays [9, 10]. Although being influenced by comorbidities and patient characteristics, Length Of hospital Stay (LOS) has been shown to be a rational choice to measure severity of disease and level of care needed in hospitalized patient [11, 12]. Therefore, we strive to evaluate cycle threshold (CT) in POC reverse-PCR tests in confirmed COVID-19 patients as possible marker for LOS and severity of disease.

Such early indicators providing insight into potential disease progression are critical in order to properly select patients requiring special care, especially when exponentially increasing numbers of infections occur in a short time interval and hospitals become overcrowded.

Not only the heterogeneous clinical presentations of COVID-19 poses a challenge for attending physicians but also the prevalence of a variety of late-effects can be difficult to manage [13]. Divided into two different entities, “post-acute COVID-19” where symptoms extend beyond 3 weeks and “chronic COVID-19” where symptoms extend beyond 12 weeks, long COVID-19 is estimated to affect up to 80% of COVID-19 patients [14, 15]. However, real-world data for hospitalized patients and long COVID-19 is limited.

## Methods

### Study design

We report data from an investigator-initiated, single-center, prospective registry study to evaluate Vivalytic™SARS-CoV-2 Multiplex POC (Bosch Healthcare Solutions GmbH; Stuttgarter, Germany) COVID-19 test in clinical practice and to evaluate CT value at admission for risk stratification in hospitalized patients conducted at the Severinenklösterchen Hospital—University of Cologne.

The protocol of this study conforms to the ethical guidelines of the 1975 Declaration of Helsinki and was approved by the institutional ethical committee of the Ärztekammer Nordrhein (20211001).

### Study population

All-comers admitted to the hospital over 18 years were eligible for inclusion. Hospital rules dictated that every patient admitted had to be tested. Tested patients were picked randomly for participation.

The decision to perform a PCR-test for SARS-CoV-2 was made independently of study inclusion by the treating physician and patients were asked to participate before the test results were available.

Written informed consent was obtained from all patients prior to study inclusion.

**Fig. 1:**
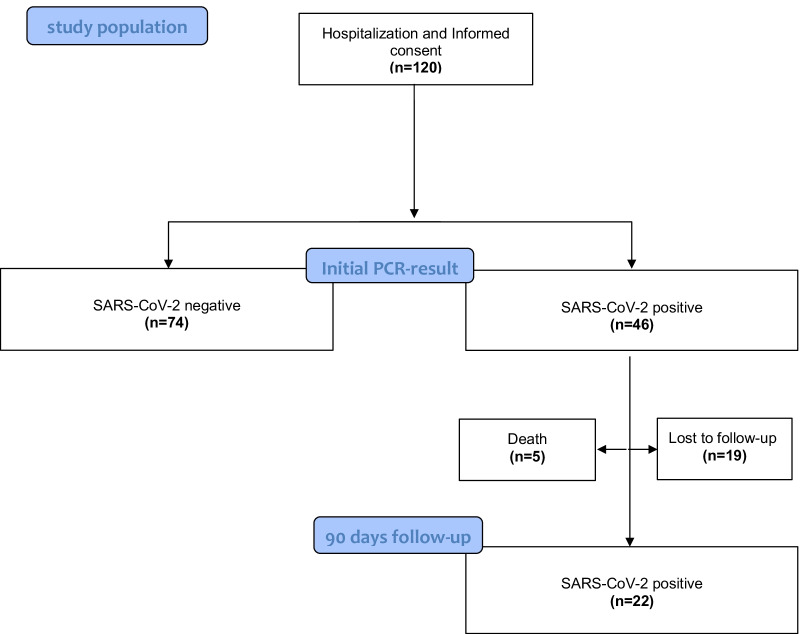
Schematic representation of the allocation of the 120 participants to the positive or negative group. Nineteen participants of the SARS-CoV-positive cohort were lost to follow-up and 5 participants died. The flow diagram is based on the template of the CONSORT flow diagram [16]

Patients with a positive PCR-test for SARS-CoV-2 were allocated to the “positive” group, patients with a negative PCR-test for SARS-CoV-2 to the control group (Fig. 1). Every patient was then tested two additional times using Vivalytic SARS-CoV-2 Test, and SARS-CoV-2 Rapid Antigen Test respectively. All nasopharyngeal swabs were taken by nurses or trained personnel.

### Study plan

Patients admitted to the hospital between January and May 2021 were randomly asked to participate in the study. If patients agreed to participate, overall characteristics such as sex, age and medical history were recorded.

To characterize the severity of the course of disease in COVID-19, patients were divided according to the length of stay (LOS): A prolonged LOS was defined as equal to or greater than the median length of hospital stay. All clinical data gathered during this period was obtained from the electronic patient file. During follow-up no interventions were applied for the purpose of this study and all therapeutic and diagnostic procedures were applied as part of standard care at the discretion of the treating physicians. Finally, participants were contacted by phone and asked about the course of disease using a standardized questionnaire after 3 months. Herein, the patients were asked to rank their current physical fitness on a scale from 0 to 100% as they felt it affected by their COVID-19 disease.

### Measurements

SARS-CoV-2 testing using Allplex™ 2019-nCOV Assay (Seegene, Seoul, Korea: polymerase chain reaction assay (PCR) targeting envelope protein- (E-), RNA-dependent RNA polymerase- (RdRP-), and N- genes) was performed by an external laboratory with Applied Biosystems™ 7500 Real-Time PCR System according to the manufacturer’s guidelines.

For analysis with Bosch Vivalytic SARS-CoV-2 assay (Bosch Healthcare Solutions GmbH, Waiblingen, Germany) using the Vivalytic SARS-CoV-2 analyzer, samples were collected in a guanidine thiocyanate-based medium (eNAT^®^ [COPAN Diagnostics Inc.; Murrieta, USA]), stabilizing the viral RNA and completely inactivating the microbial viability. Vivalytic SARS-CoV-2 analyzer is a portable device and works fully automatically.

Roche SARS-CoV-2 Rapid Antigen Test is an immunochromatographic assay for qualitative detection of SARS-CoV-2 infection in nasopharyngeal swabs. The presence of viral antigens in sufficient concentration enables binding to specific mouse monoclonal anti-SARS-CoV-2 antibodies, which is then reflected by the appearance of a visual indicator after the clinical specimen is collected and then deposited in a pre-filled extraction buffer container with the solution being put on the test sample according to manufacturer’s guidelines.

Inter-assay variability was performed conducting repetitive tests on four samples throughout 3 days in triplicates. Intra-assay variability was performed conducting four consecutive assays in duplicates in one run. For intra assay precision we used inactivated whole pathogen sample (AMPLIRUN^®^ TOTAL SARS-CoV-2 RNA Control [Vircell SA, Granada, Spain]) and for inter assay precision we used both, inactivated whole pathogen sample as well as patient samples. 15 000 AMPLIRUN^®^ SARS-CoV-2 RNA CONTROL copies were reconstituted in guanidine thiocyanate-based medium eNAT^®^ (COPAN Diagnostics Inc.; Murrieta, USA) to generate the control sample. Then a dilution series of consecutive samples was performed (1:2; 1:10; 1:100; 1:1000). Both, the results from Bosch Vivalytic (Bosch Healthcare Solutions GmbH; Stuttgarter, Germany) SARS-CoV-2 test for Bosch Vivalytic One as well as the results from the COVID-19 ELISA Elecsys Anti-SARS-CoV-2 assay (Roche Holding AG; Basel, Swiss) were compared to the results from the current gold standard at Severinenklösterchen Hospital – University of Cologne, a centralised laboratory PCR test (Seegene [Seegene, Inc; Seoul, South Korea] AllplexTM2019-nCoV) carried out by an extern laboratory (Laboratory Dr. Wisplinghoff, Horbeller Str. 18–20 Koeln, Germany) to calculate inter- and intra- assay precision.

### Statistic

Continuous patient data were compared using a Mann–Whitney U-test or unpaired t-test. Categorical differences between patient groups were compared using Fishers exact test. Continuous variables are presented as mean ± standard deviation (SD) if found to follow a Gaussian distribution according to the D’Agosstino-Pearson omnibus normality test or as median ± lower and upper quartiles if found to follow a non-Gaussian distribution. Categorical patient characteristics are presented as percentages. Spearman nonparametric correlation was used to evaluate the degree to which LOS and CT value at admission move in relation to each other. Independent predictors of LOS above or below median length of stay (Temperature at admission [°C], hemoglobin [g/dl], CT value, thrombocytes [K/μl], sex, age, C-reactive protein [mg/dl], abnormal chest image at admission, Lactate dehydrogenase [U/I], Aspartate Aminotransferase [U/I], Leukocytes [K/μl]) were investigated using multivariate logistic regression.

Descriptive analyses were performed using Graph Pad Prism Version 9.0 (Prism 9 for Mac OS X; GraphPad Software. Inc. La Jolla. CA).

## Results

### Baseline characteristics

We included 120 patients hospitalized between January and May 2021 in this prospective cohort study. Of those, 46 (38%) patients tested positive for COVID-19 infection via the external laboratory. The COVID-19 positive group was younger when compared to the negative control group (55.5 [38.5–65.75] years vs. 63.05 [± 17.41] years; p = 0.007).

Patients with COVID-19 disease had higher levels of Lactate dehydrogenase (LDH) (362.3 [± 136.1] U/I vs. 236 [203.3–301]; p = 0.014) and C-reactive protein (CRP) (5.96 [± 5.348] mg/dl vs. 0.4 [0.4–2.7] mg/dl; p < 0.001) when compared to the control group. If the cut-off for a normal value for LDH was considered to be 250 U/I, an increased level of LDH at baseline had a sensitivity of 75% and a specificity of 53.5% (PPV 50%; NPV 77.5%) for COVID-19 diagnosis. There was no difference with anamnesis for coronary artery disease (8 [17.4%] patients vs. 15 [20.3%] patients; p = 0.495), hypertension (18 [39.1%] patients vs. 36 [48.6%] patients; p = 0.349) or pulmonary disease (9 [19.5%] patients vs. 25 [33.9%] patients; p = 0.101). Symptoms of COVID-19 patients included dyspnea (39.1%), cough (30.4%), fatigue (37%), gastrointestinal complaints (17.4% [Abdominal pain, diarrhea and anosmia]), fever (41.3%) and chest pain (8.7%). The majority of COVID-19 patients were affected by the virus variant B1.1.7 (31 [67.4%] of all included COVID-19 patients) (Table 1).

**Table 1:** Patients baseline characteristics at admission

Variable	COVID-19 n = 46		Non-COVID-19 n = 74		P-value
Age (years)	55.5	38.5–65.75	63.05	± 17.41	0.006^a^
Sex (female)	15	(33.3%)	39	(52.7%)	0.057^a^
C-reactive protein (mg/dl)	5.96	(± 5.348)	0.4	(0.4–2.7)	< 0.001^b^
Hemoglobine /g/dl)	13.46	(± 2.068)	12.79	(± 2.164)	0.094^b^
Leukocytes (K/μl)	6.75	(4.575–9.85)	7.9	(6.15–10.2)	0.169^b^
Platlets (K/μl)	217.5	(179–261.8)	258.5	(195.3–310.3)	0.116^b^
Lactate dehydrogenase (U/I)	362.3	(± 136.1)	236	203.3–301	0.014^b^
Aspartate Aminotransferase (U/I)	38	(26.5–55)	24	17–31.25	0.614^b^
CAD*	8	(17.4%)	15	(20.3%)	0.495^c^
Hypertension	18	(39.1%)	36	(48.6%)	0.349^c^
Pulmonary disease	9	(19.5%)	25	(33.9%)	0.101^c^
Length of Stay (days)	9.5	(4.75–15.25)			

*CAD = Coronary Artery Disease; Continuous variables are presented as mean ± standard deviation (SD) if found to follow a Gaussian distribution according to the D’Agosstino-Pearson omnibus normality test or as median ± lower and upper quartiles if found to follow a non-Gaussian distribution.; ^a^Nonparametric Mann–Whitney U test to compare ranks; ^b^Unpaired t-test; ^c^Fisher’s exact test

### Bosch Vivalytic SARS-CoV-2 assay precision

Inter- and Intra-assay coefficients of variability for Vivalytic™SARS-CoV-2 Multiplex analyzer were below 15% (Tables 2, 3, 4).

**Table 2:** Inter-test variability for different test samples in different dilution steps using inactivated whole pathogen sample

Sample dilution factor	CT-value	CT-value	CT-value	Mean	SD^*^	CV^**^ (%)
1:2	31.9	31	33	31.97	1.00	3.1
1:1	30.5	31.8	31.9	31.4	0.78	2.5
1:100	31.7	34.9	31.5	32.7	1.91	5.8
1:1000	33.7	38.3	36.7	36.23	2.33	6.4

*SD = Standard Deviation; **Coefficient of Variation

**Table 3:** Intra- variability for different test samples in different dilution steps using inactivated whole pathogen sample

Sample dilution factor	CT-value	CT-value	Mean	SD^*^	CV^**^ (%)
1:2	34.1	33	33.55	0.78	2.32
1:1	32.6	31.9	32.25	0.49	1.52
1:100	38.1	31.5	34.8	4.67	13.41
1:1000	35.4	36.7	36.05	0.92	2.55

*SD = Standard Deviation; **Coefficient of Variation

**Table 4:** Inter-assay test variability for different test samples in different dilution steps using patient samples

Sample dilution factor	CT-value	CT-value	CT-value	Mean	SD*	CV^**^ (%)
1:2	29.2	28.5	29.3	29	0.44	1.5
1:1	30.3	27.5	32.1	29.97	2.32	7.74
1:100	27.8	31.3	28.5	29.2	1.85	6.34
1:1000	29	31.9	35.5	32.13	3.26	10.13

*SD = Standard Deviation; **Coefficient of Variation

In relation to the results of the external laboratory RT-PCR test, the POC test had a test-sensitivity for SARS-CoV-2 infection of 88% with a specificity of 96%. The positive predictive value was 90% and the negative predictive value 95%.

The antigen test had a sensitivity for COVID-19 infection of 65% with a specificity of 100%. The positive predictive value for the antigen test was 100% and the negative predictive value 88% when compared to the AllplexTM2019-nCoV assay.

Computing a nonparametric spearman correlation showed a high correlation between the CT Values from the Vivalytic SARS-CoV-2 assay and the AllplexTM2019-nCoV assay (r[38]  = 0.6158, p ≤ 0.0001). To further assess the agreement between the two methods, Bland–Altman plot was used. After eliminating CT values above the maximum number of cycles (CT < 35; N = 16), Bland-Atman shows good agreement between both test methods (limits of agreement 0.2125 [− 1,777,113–1,819,613]).

### Length of stay

Patients with positive SARS-CoV-2 tests had an overall median length of hospital stay of 9.5 (4.75–15.25) days. To quantify severity of disease we divided COVID-19 patients into two groups. A prolonged LOS was defined as equal to or greater than the median length of hospital stay [Patients with LOS ≥ 10 days (21 [± 17] days) vs. patients with LOS < 10 days (4.7 [± 2.7] days)].

**Fig. 2:**
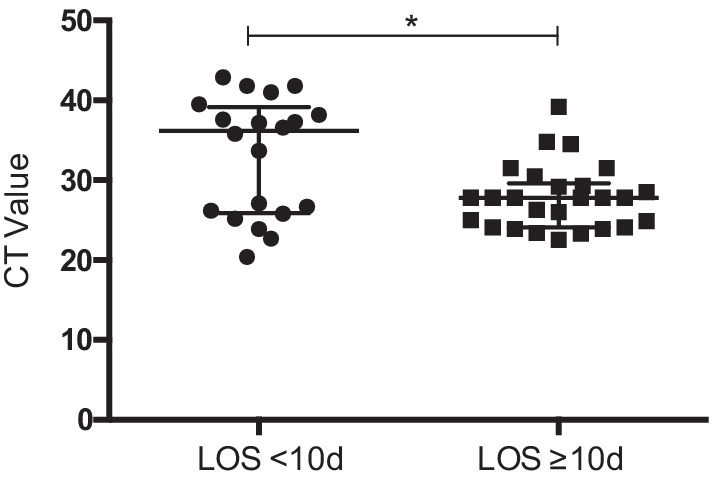
Point-of-Care cycle- threshold (CT) Value at admission in patients with confirmed diagnose of COVID-19 and length of hospital stay (LOS) above or below the median LOS stay respectively. Data are presented as scatter block with Interquartile Range

Patients with LOS < 10 days had significantly lower CT values at admission when compared to patients with LOS ≥ 10 days (36.2 [25.9–39.18] vs. 27.82 [± 4.648]; p = 0.0191). (Fig. 2) In a multiple logistic regression analysis with the dichotomous outcome being LOS < 10 days vs. LOS no ≥ 10 days, age was the only factor associated with LOS (OR 1.105 [95% CI, 1.03–1.19]; p = 0.006).

Patients with a LOS < 10 days were younger (45.6 [± 14] years vs. 62.4 [± 62.4] years; p = 0.001) and had higher levels of haemoglobin (13.6 (± 1.7) g/dl vs. 11.9 (± 2.3) g/dl; p = 0.007) when compared to patients with a LOS > 10 days. There was a negative correlation of CT at admission and LOS (r[44]_s_ = − 0.31; p = 0.038). There was no statistically significant difference between both groups regarding other baseline characteristics collected. In the group with LOS < 10 days, 15 (75%) patients were affected by the virus variant B1.1.7 and there were 16 (61.5%) patients affected in the group with LOS ≥ 10 days (p = 0.4834). (Table 5).

**Table 5:** Baseline characteristics at admission in patients hospitalized with COVID-19 disease with length of hospital stay (LOS) above or below 10 days respectively

Variable	LOS < 10 days n = 23		LOS ≥ 10 days n = 23		P-value
LOS*	4.7	(± 2.7)	21	(± 17)	
Age (years)	45.6	(± 14)	62.4	(± 62.4)	0.001^b^
Sex (female)	7	(30.4%)	8	(34.8%)	0.753^c^
C-reactive protein (mg/dl)	3.6	(0.4–7.6)	7.7	(± 6.3)	0.051^b^
Haemoglobine (g/dl)	13.6	(± 1.7)	11.9	(± 2.3)	0.007^b^
Leukocytes (Tsd/ul)	7.5	(± 4)	7.4	(4.9–10)	0.628^a^
Platlets (Tsd/ul)	209	(173–257)	233	(181—282)	0.652^a^
Lactate dehydrogenase (U/I)	330	(258–469)	377	(230–447)	0.989^a^
Aspartate Aminotransferase (U/I)	38	(16–38)	46	(± 30)	0.941^a^
CAD**	2	(9%)	6	(25%)	0.119^c^
Hypertension	7	(29%)	11	(48%)	0.567^c^
Pulmonary disease	4	(17%)	5	(22%)	0.710^c^
Virus Variant B1.1.7	15	(65%)	11	(48%)	0.234^c^
POC-CT*** at admission	36.2	(25.9–39.18)	27.82	(± 4.648)	0.019^a^
Vital parameters at admission					
Mean Pressure (mmHg)	94.5	(81–105)	90	(87–94)	0.616^a^
Temperature	37.6	(36.8–38.5)	37.4	(36.5–38.1)	0.410^a^
Respiratory Rate (per min.)	18.3	(± 4.8)	17.6	(± 3.2)	0.530^b^
Heart Rate (Beats/minute)	87.7	(± 15.1)	92.4	(± 18.7)	0.367^b^
Saturation at admission (%)	96	(94.8–98)	95.5	(89.5–96.3)	0.127^a^

*LOS = Length of stay; **CAD_ Coronary Artery Disease; ***CT = Cycle Threshold only CT values of the Point-of Care test (LOS < 10 days n = 20 and LOS ≥ 10 days n = 20); Continuous variables are presented as mean ± standard deviation (SD) if found to follow a Gaussian distribution according to the D’Agosstino-Pearson omnibus normality test or as median ± lower and upper quartiles if found to follow a non-Gaussian distribution. ^a^nonparametric Mann–Whitney U test to compare ranks; ^b^unpaired t-test; ^c^Fisher’s exact test

### Outcome

Of 46 patients who tested positive, 22 completed the follow-up and 5 COVID-19 patients died (4 patients due to COVID-19 on day 19, day 5, day 14 and day 5 after inclusion and 1 patient due to severe heart failure). All patients died while being hospitalized.

At 3 months after hospital discharge, 15 (68%) COVID-19 patients had persistent depreciation of quality of life incidental with COVID-19 disease (80 [70–90] % of the normal physical capacity).

## Discussion

In this single centre prospective cohort study we evaluate real-world diagnostic accuracy of point-of-care testing using Bosch Vivalytic SARS-CoV-2 test for Bosch Vivalytic One and a commercially available antigen test by comparing sensitivity and specificity to an established RT-PCR test performed by an external laboratory.

Our prospective and comparative evaluation shows that Bosch Vivalytic SARS-CoV-2 POC test is a valid strategy for COVID-19 detection with the potential to decrease door-to-diagnosis-time allowing earlier detection of COVID-19 positive patients [3, 17]. With its advertised turnaround time of approximately 39 min it far exceeds those of established external laboratories using RT-PCT by far [5]. The agreement of the POC test and RT-PCR is good with robust inter- and intra-assay reliability and good detection of positive samples. However, recently published data indicates that in the very early phase of the infection with high CT values, in which the RT-PCR can already detect the infection, the antigen level is not high enough for the POC test, necessitating repeated testing [18, 19].

While RT-PCR should still be the gold standard, POC testing could help with patient triage and improve patient flow dynamics [20]. The considerably higher sensitivity of the POC test when compared to antigen detection alone especially qualifies it to reduce the number of missed infections with COVID-19, a serious risk when using the antigen test as reported in other publications [21].

The prognostic value of CT in COVID-19 patients has been suggested by several studies [22]. Prospective clinical data as well as data depending on CT values acquired using POC tests however is scarce. Our data shows that on admission POC CT values are lower in patients with prolonged length of hospital stay for COVID-19 patients and that CT value at admission correlates with LOS. In a multivariate analysis however, only age was associated with LOS and variability of CT values of up to 8 cycles were observed for the same specimen material tested in a different laboratory in a recent publication [23]. Therefore, risk assessment using CT values should only be considered if tests are acquired from only one laboratory using the same assay and can only add to initial risk assessment.

Nevertheless, through their easier implementation, cost effectiveness and accuracy POC COVID-19 test could help monitoring epidemiological features of the COVID-19 pandemic through testing of large cohorts [24].

Long-term health effects of COVID-19 called Long-COVID or COVID long-haulers are challenging for clinicians through their heterogeneity [13]. It is vital to understand the percentage of patients suffering from long-term effects. Our data shows, that up to 68% of discharged patients experience a COVID-19 related decrease in quality of life at three months after hospital discharge. This is in line with several other studies and the recommendation for patients discharged after COVID-19 hospitalization to follow-up with their primary care physician on a regular basis [25, 26].

Our data depicts the third wave of the COVID-19 pandemic in Germany early 2021 with SARS-CoV-2 Variant B.1.1.7 being the predominantly detected variant [27]. This mutant strain prompted border closures, lockdowns and new restrictions ultimately cumulating in further deaths and economic loss. However, with a large proportion of the elderly being already inoculated following the guidelines of the Standing Committee on Vaccination (STIKO) we report COVID-19 patients to be younger then regular hospital all-comers forming our control group [28]. This all-the-more emphasises the need for rapid inoculation to protect patients.

Several routine laboratory markers have been suggested in the COVID-19 diagnostic algorithm [29]. In a recent meta-analysis LDH has been suggested as a cost-effective biomarker in patients with COVID-19 [30]. An important variable in our study is that two distinct patient groups were analysed one composed from regular all-comers and one of COVID-19 patients. In our prospective data, elevated LDH was a distinct feature of COVID-19 patients when compared to the control group. However, LDH was not associated with LOS. Larger clinical trials are needed to confirm the value of LDH at baseline for COVID-19 diagnosis.

### Limitations

POC PCR is a qualitative not quantitative measurement and it is not possible to quantify the virus load via CT values. CT values vary in-between different assays. We used only a single negative RT-PCR to confirm the absence of COVID-19 infection, risking missing infection. A lot of patients were lost to follow-up mainly due to the quantity of measures taken during the pandemic and the hence decreasing will of people to participate. We did not assess quality of life at the time point of inclusion. The fact, that patients with LOS > 10 days were older could possibly diminish the interpretability of our data. Multivariate analysis showed only age being associated with LOS. However, we argue, that within the complex pathology that is COVID-19, CT value could be a valid tool to identify patients at high risk. Larger clinical trials are needed to validate this.

## Conclusion

This data from a single centre provides prospective and comparative data indicating, that POC testing using Bosch Vivalytic SARS-CoV-2 is a valid strategy to identify COVID-19 patients and decrease turnaround time during COVID-19 pandemic. Also, our data suggets that the on admission CT value, maybe combined with on admission age, might be a marker for length of hospital stay and severity of disease in COVID-19 patients. Our data emphasise the increased risk for long-term health effects of COVID-19 patients and the consecutive need for individual follow-up.

## Data Availability

The datasets analysed during the current study are available from the corresponding author on reasonable request.

## Abbreviations

COVID-19: Coronavirus disease 2019
POC: Point of care
CT: Cycle threshold
LOS: Length of hospital stay
SARS-CoV-2: Severe acute respiratory syndrome coronavirus 2
